# Tocopherols and Antioxidant Potential of Rapeseed Pomace: A Multi-Method Evaluation for Food and Feed Applications

**DOI:** 10.3390/molecules30224405

**Published:** 2025-11-14

**Authors:** Bronisław K. Głód, Mateusz Borkowski, Jolanta Marciniuk, Paweł Marciniuk

**Affiliations:** 1 Institute of Chemical Sciences, Faculty of Natural Sciences, University of Siedlce, 3 Maja St. 54, 08-110 Siedlce, Poland; 2 Łukasiewicz Research Network-Industrial Chemistry Institute, Rydygiera St. 8, 01-793 Warsaw, Poland; 3 Institute of Biological Sciences, Faculty of Natural Sciences, University of Siedlce, Bolesława Prusa St. 14, 08-110 Siedlce, Poland; jolanta.marciniuk@uws.edu.pl (J.M.); pawel.marciniuk@uws.edu.pl (P.M.)

**Keywords:** antioxidant capacity, rapeseed pomace, electrochemical detection

## Abstract

Rapeseed pomace, a by-product of oil processing, is a rich source of bioactive compounds with potential antioxidant properties. This study aimed to evaluate the tocopherol content and antioxidant capacity of rapeseed pomace in comparison with commonly used edible oils. Chromatographic analysis using high-performance liquid chromatography with electrochemical detection (HPLC–ED), spectrophotometric assays including 2,2-diphenyl-1-picrylhydrazyl (DPPH) radical scavenging and the Folin–Ciocalteu method, as well as electrochemical approaches such as cyclic voltammetry (CV) and differential pulse voltammetry (DPV), were applied to provide a comprehensive assessment. Rapeseed pomace exhibited the highest total tocopherol concentration (>70 mg/100 g) and strong antioxidant activity confirmed across all assays. Significant correlations (r ≈ 0.9) between spectrophotometric and electrochemical results demonstrated the reliability of the applied techniques. In addition, methanol extraction was applied as an alternative to saponification, providing a straightforward approach for tocopherol determination. The findings highlight rapeseed pomace as a valuable natural antioxidant source and support the applicability of electrochemical methods for the evaluation of edible oils.

## 1. Introduction

Edible oils play a crucial role in human nutrition [1], serving not only as a source of essential fatty acids [2] but also as carriers of natural and synthetic bioactive compounds with antioxidant properties, which contribute to their stabilization [3,4,5,6]. Antioxidants present in edible oils mitigate oxidative stress [7,8], a condition linked to various pathologies, including cardiovascular diseases [9] and cancer [10]. The antioxidative capacity of oils is primarily attributed to bioactive constituents such as tocopherols [11], polyphenols [12], and other secondary metabolites [3]. The antioxidative potential varies among different edible oils, contingent upon their compositional profiles. For example, extra virgin olive oil is particularly abundant in phenolic compounds, notably hydroxytyrosol [13,14] and oleuropein [15], which substantially enhance its oxidative stability and confer health benefits. Likewise, flaxseed [16], rapeseed [12], and sesame oil [17] are characterized by high concentrations of lignans and other antioxidants that influence both their oxidative stability and nutritional value.

Moreover, antioxidants may interact synergistically or antagonistically, thereby influencing the overall antioxidant capacity of the oil sample. Consequently, it is preferable to assess the Total Antioxidant Potential (TAP), which reflects the combined effect of all antioxidants present, factoring in both their total concentration and their reaction rates with the radicals employed in the assay [18]. Although various terms have been used in the literature to describe this parameter [19,20], the term TAP is adopted herein for consistency.

Several analytical techniques have been employed to assess the antioxidative properties of edible oils. These methods are commonly classified based on their underlying reaction mechanisms into hydrogen atom transfer (HAT), single electron transfer (SET), and mixed-mode assays, where both mechanisms occur simultaneously [21,22]. HAT-based methods include the Oxygen Radical Absorbance Capacity (ORAC) assay, which utilizes reagents such as Azobisisobutyronitrile (AIBN), 2,2′-Azobis(2-amidinopropane) dihydrochloride (ABAP), 2,2′-Azobis(2,4-dimethylvaleronitrile)(AMVN), and 2,2′-Azobis(2-methylpropionamidine) dihydrochloride) (AAPH) [23,24], SET-based methods comprise assays such as Cupric Ion Reducing Antioxidant Capacity (CUPRAC) [25], Ferric Reducing Antioxidant Power (FRAP) [26] and the Folin–Ciocalteu (FC) assay [27]. Mixed-mode (SET/HAT) assays include 2,2-diphenyl-1-picrylhydrazyl (DPPH) [28], 2,2′-Azino-bis(3-ethylbenzothiazoline-6-sulfonic acid) (ABTS) [24], Trolox Equivalent Antioxidant Capacity (TEAC) [29] and N,N-dimethyl-p-phenylenediamine (DMPD) [30]. A secondary classification can be based on the instrumentation employed, grouping methods into photometric, electrochemical, and chromatographic techniques. The present study focuses on the following methods: DPPH and Folin–Ciocalteu assays (for total phenolic content), cyclic voltammetry (CV), and high-performance liquid chromatography with electrochemical detection (HPLC–ED). DPPH is a widely utilized SET-based assay that measures the capacity of antioxidants to reduce the DPPH radical, leading to a decrease in absorbance at 517 nm [31]. This assay provides an estimation of the free radical scavenging activity of antioxidants present in the oil [32]. The Folin–Ciocalteu assay, also SET-based, quantifies the total phenolic content by forming a blue complex upon reaction of phenolic compounds with the reagent, which is then measured spectrophotometrically at 765 nm. Although the assay lacks specificity for phenolics, it remains extensively used for evaluating antioxidative potential [33]. Cyclic voltammetry is an electrochemical technique that offers insights into the redox properties of antioxidants. By applying a sweeping potential, it enables characterization of the electrochemical behavior of antioxidant compounds [34,35] making it particularly suitable for analyzing complex oil matrices [36]. HPLC–ED combines chromatographic separation with electrochemical detection, facilitating the precise quantification of individual antioxidants in edible oils. This method exhibits high sensitivity and selectivity, rendering it a valuable tool for detailed antioxidative profiling [37].

Numerous studies have examined the antioxidative properties of edible oils employing diverse analytical methodologies. It has been demonstrated that oils enriched with polyphenols and tocopherols possess enhanced antioxidative capacity [38]. For instance, comparative analyses of various edible oils using DPPH and Folin–Ciocalteu assays have revealed a strong correlation between phenolic content and radical scavenging activity [39]. Electrochemical investigations have further substantiated the significant role of these compounds in conferring oxidative stability. Additionally, HPLC–ED analyses have indicated that the antioxidative efficacy of oils is modulated by processing techniques, storage conditions, and synergistic interactions among antioxidants [40,41].

Despite considerable progress in evaluating the antioxidative properties of edible oils, selecting an appropriate analytical method remains challenging. Different techniques yield distinct insights into antioxidant activity due to variations in reaction mechanisms, detection principles, and compound-specific sensitivities. Electrochemical approaches, such as CV and differential pulse voltammetry (DPV), enable direct redox characterization of antioxidants; however, their interpretation within complex matrices is often complicated. Spectrophotometric assays, including DPPH and Folin–Ciocalteu methods, provide estimates of the overall antioxidant potential but can be affected by interfering substances and the nature of the radicals employed. HPLC–ED facilitates the separation and quantification of individual antioxidants, though its correlation with total antioxidant capacity remains ambiguous [34,42,43]. Given these methodological disparities, a systematic comparison of these techniques is necessary to assess their reliability, sensitivity, and suitability for edible oil analysis. Recent literature emphasizes the necessity of detecting antioxidant activity in edible oils to better ensure their oxidative stability and quality during storage [44]. Furthermore, standardization of protocols and optimization of electrochemical methods could enhance the accuracy and reproducibility of antioxidant evaluations. This study seeks to address this gap by evaluating photometric, electrochemical, and chromatographic techniques for the determination of TAP, comparing their respective advantages and limitations in the context of food analysis.

Rapeseed pomace, a by-product of rapeseed oil production, is seldom utilized in household applications. This study investigated its antioxidant properties by assessing (i) tocopherol concentrations and (ii) TAP. TAP was determined using cyclic voltammetry and amperometric detection coupled with HPLC. These results were compared to indirect methods measuring TAP via DPPH radical scavenging activity and total phenolic content. The findings suggest that rapeseed pomace contains the highest antioxidant concentrations, including tocopherols. Among the edible oils analyzed, rapeseed oil exhibited the greatest antioxidant content, whereas olive oil showed relatively lower levels. Notably, TAP values obtained through cyclic voltammetry differed from those of other methods, as this technique also accounted for weaker antioxidants. Due to the elimination of extensive sample preparation steps, such as saponification and the relative simplicity of measurement and signal processing, the HPLC method with amperometric detection was identified as the most advantageous.

The growing interest in functional foods and nutraceuticals has driven research into natural sources of antioxidants. Edible oils and oilseed byproducts, such as rapeseed pomace, are rich in bioactive compounds with potential health benefits. Rapeseed pomace, a byproduct of oil extraction, contains phenolic compounds, tocopherols, and other antioxidants. While the antioxidant properties of edible oils are well studied, there is limited comparative analysis between rapeseed pomace and commercial oils. Comparing these matrices may help evaluate the potential valorization of rapeseed pomace in food, feed, or supplement applications, and determine whether its antioxidant properties can compete with or complement those of commercial oils.

Recent literature also highlights increasing interest in revalorizing agro-industrial byproducts. For instance, a 2025 article provided an integrated evaluation of grape pomace from multiple varieties (2022–2024), profiling its fatty acid, total phenolic, flavonoid, and antioxidant parameters (DPPH, ABTS, ORAC), and demonstrating its suitability for functional, nutraceutical use [44,45]. In another study, incorporation of tomato pomace into sunflower and rapeseed oils significantly improved oxidative and thermal stability and enriched the oils with bioactive compounds such as carotenoids [46]. Moreover, polyphenolic extracts from rapeseed pomace, particularly sinapic acid, possess strong antioxidant activity, supporting further valorization of rapeseed processing residues [47].

The novelty of this study lies in two aspects: first, the use of methanol extraction as a rapid and cost-effective alternative to classical saponification for HPLC analysis of tocopherols; and second, the integration of chromatographic, spectrophotometric, and electrochemical assays to evaluate the TAP of both edible oils and rapeseed pomace. These innovations enable a more comprehensive and comparative assessment of antioxidant profiles in oil matrices and by-products.

Therefore, the aim of this study was to comprehensively evaluate the tocopherol content and antioxidant potential of rapeseed pomace in comparison with selected edible oils, using a multi-method approach that combines chromatographic (HPLC–ED), spectrophotometric (DPPH and Folin–Ciocalteu), and electrochemical (CV and DPV) techniques.

## 2. Results and Discussion

### 2.1. Methanol Extraction Evaluation

**Figure 1:**
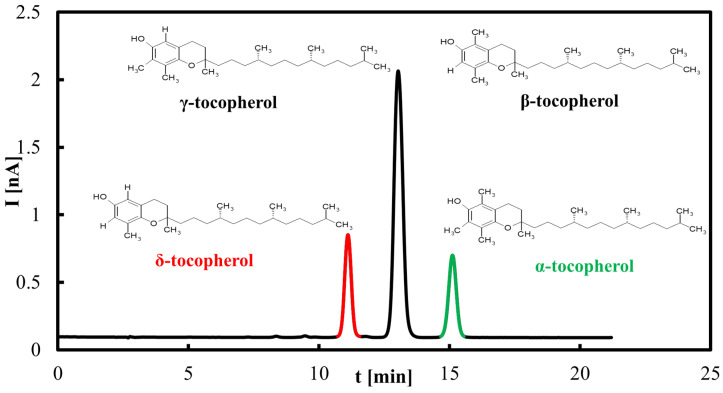
Representative HPLC–ED chromatogram of tocopherols standard. Peaks correspond to individual tocopherol homologues (*α*, *β+γ*, *δ*).

The saponification procedure (according to the PN-EN 12822 standard [48]) is time-consuming, requiring 60 to 90 min, and is not cost-effective. Therefore, an alternative method based on the extraction of oils into methanol using an ultrasonic bath was developed. This approach reduces sample preparation time to approximately 25 min using 10 mL of methanol. The results presented below were obtained using this methanol extraction method. Figure 1 shows the chromatogram of the tocopherol standards. It was observed that β- and γ-tocopherols were not separated under the developed chromato-graphic conditions.

**Figure 2:**
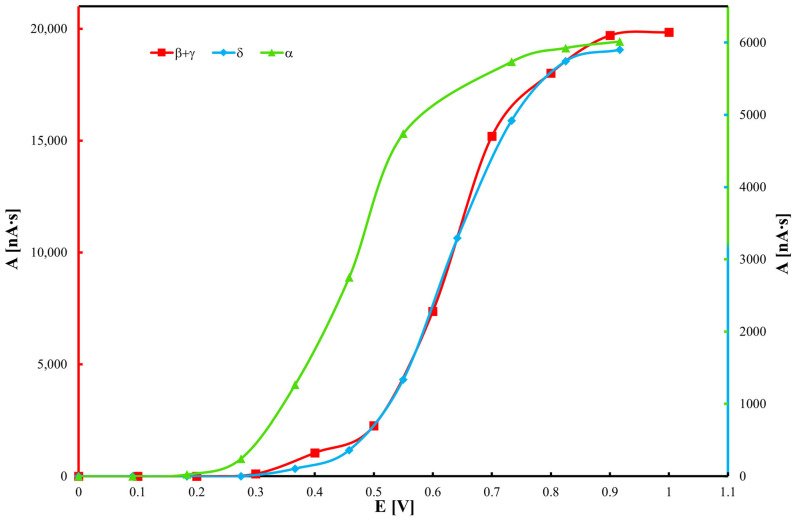
Hydrodynamic voltammograms of tocopherol homologues obtained by electrochemical detection (ED).

Hydrodynamic voltammograms of tocopherols, derived from chromatographic data, are presented in Figure 2. Results indicate that α-tocopherol exhibits the strongest antioxidant activity, as it is oxidized at the lowest potential, consistent with previous voltammetric studies [49,50]. Each tocopherol displays three distinct voltammetric peaks. An applied potential of 0.8 V was found to be optimal, producing the highest chromato-graphic peak intensities. Increasing the potential beyond this value resulted in elevated noise levels without enhancing peak heights. Notably, water electrolysis occurs at potentials above 1.3 V on the glassy carbon electrode. The detection limit for α-tocopherol was determined to be 2 μM.

**Figure 3:**
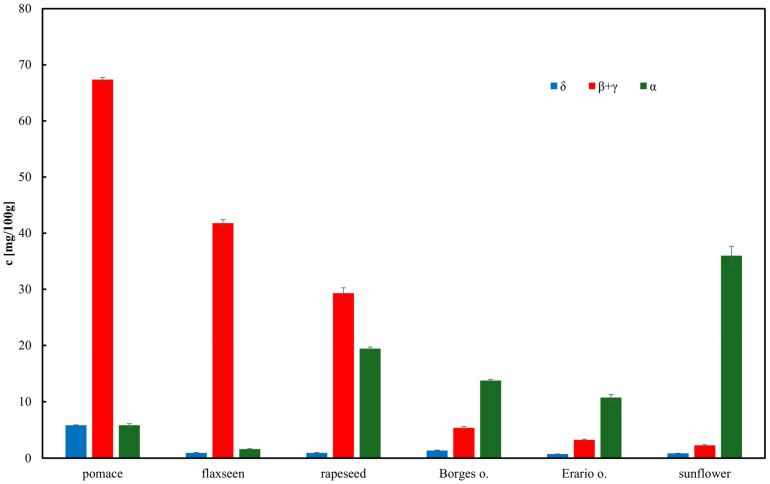
Content of tocopherol homologues in edible oils. Results are expressed as mean ± SD (*n* = 3).

**Figure 4:**
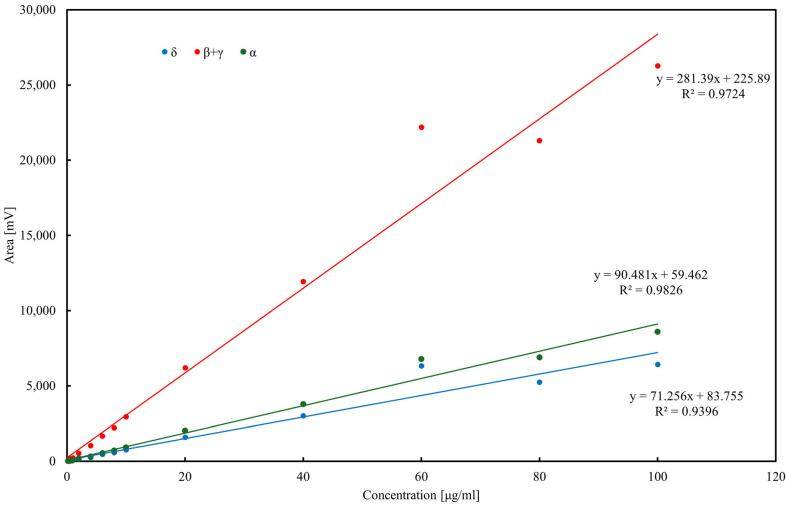
Calibration curves of tocopherol homologues determined by HPLC–ED.

The developed assay was applied to determine tocopherol concentrations in various oils. The total tocopherol content in the samples is presented in Figure 3. The highest tocopherol concentration was observed in rapeseed pomace, comparable to that found in rapeseed oil. This suggests that a substantial portion of tocopherols, except for α-tocopherol, remains in the pomace following oil pressing. Sunflower oil exhibited the highest concentration of α-tocopherol, consistent with previous reports [51,52]. Notably, olive oil contained relatively low total tocopherol levels, with α-tocopherol as the pre-dominant isoform [53]. This observation is further supported by reports showing α-tocopherol concentrations in olive oils in the range of 40–60 µg/mL, consistent with our findings (<50 µg/mL) [39]. The chromatographic procedure for tocopherol determination was validated using external calibration in the range 0.2–100 µg/mL (Figure 4). Linear regression showed satisfactory correlation coefficients (*R*^2^ = 0.97 for δ-tocopherol, 0.99 for β+γ-tocopherols, and 0.99 for α-tocopherol). The calculated limits of detection (LOD) were 1.9 µg/mL for α-tocopherol, 2.3 µg/mL for β+γ-tocopherols, and 3.5 µg/mL for δ-tocopherol, while the corresponding limits of quantification (LOQ) were 5.8, 7.1, and 10.7 µg/mL, respectively. Repeatability of the method, evaluated by replicate injections at 10 µg/mL, was within 3% relative standard deviation (RSD) for peak areas and below 0.5% RSD for retention times. Although δ-tocopherol exhibited slightly weaker linearity at low concentrations, overall method performance confirmed the reliability of HPLC–ED for quantitative analysis of tocopherols in edible oils and rapeseed pomace.

### 2.2. TAP^HPLC^ Measurements

**Figure 5:**
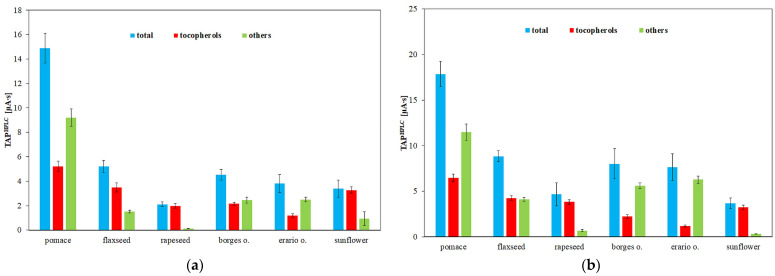
Total antioxidant potential determined by HPLC–ED (TAP^HPLC^) in edible oils obtained at (**a**) 0.6 V and (**b**) 0.8 V. Results are expressed as mean ± SD (*n* = 3).

**Figure 6:**
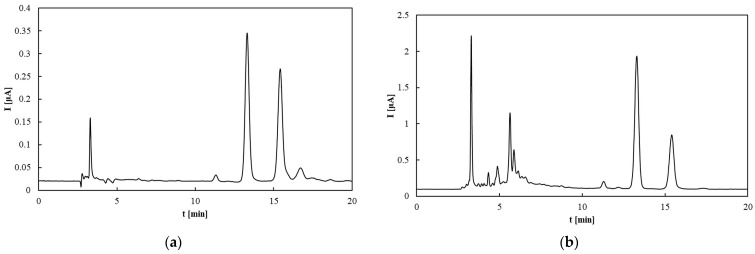
HPLC–ED chromatograms of rapeseed pomace at (**a**) 0.6 V and (**b**) 0.8 V.

Antioxidants can be detected electrochemically via their oxidation at positive potentials, allowing their quantification using an electrochemical detector following chromatographic separation. An alternative TAP assay based on measuring the total area of all chromatographic peaks recorded by the amperometric detector (TAP^HPLC^) was proposed [42]. In this study, measurements were performed at two different working electrode potentials (Figure 5). At the lower potential (0.6 V), only strong antioxidants are detected, whereas at the higher potential (0.8 V), both strong and weak antioxidants contribute to the signal. The chromatograms recorded at these potentials (Figure 6) reveal that increasing the potential not only elevates the peak heights but also results in the appearance of new peaks corresponding to weaker antioxidants. Rapeseed pomace exhibited the highest TAP value, which aligns with the high tocopherol content shown in Figure 3. Notably, the TAP of rapeseed oil and its pomace is largely attributed to tocopherols. Conversely, the antioxidant potential in flaxseed pomace is influenced more by other compounds such as phytosterols, phospholipids, and phenolic acids (e.g., p-hydroxybenzoic, salicylic, and cinnamic acids) [54]. Flaxseed oil contains a significant amount of weak antioxidants, as evidenced in Figure 5a,b, consistent with previous findings [55]. For olive oils and flaxseed oil, the predominant antioxidants are relatively weak, as indicated by the noticeable increase in TAP values between 0.6 and 0.8 V. The main difference between these oils is their tocopherol content, which is lower in Borges and Erario olive oils, consistent with the HPLC tocopherol analysis shown in Figure 5. Sunflower oil displays TAP values similar to rapeseed oil, where total tocopherol content strongly influences antioxidant properties despite differences in tocopherol forms, consistent with previous findings [47,56].

Overall, a strong correlation was observed between TAP^HPLC^ and the total tocopherol concentration, underscoring the importance of tocopherols in the antioxidant capacity of edible oils. 

### 2.3. TAP^DPPH^ Measurements

DPPH is a stable free radical that primarily reacts with strong antioxidants [57]. Consistent with the other analytical methods, the highest TAP^DPPH^ values were observed for rapeseed pomace, while sunflower oil exhibited the lowest antioxidant potential.

**Figure 7:**
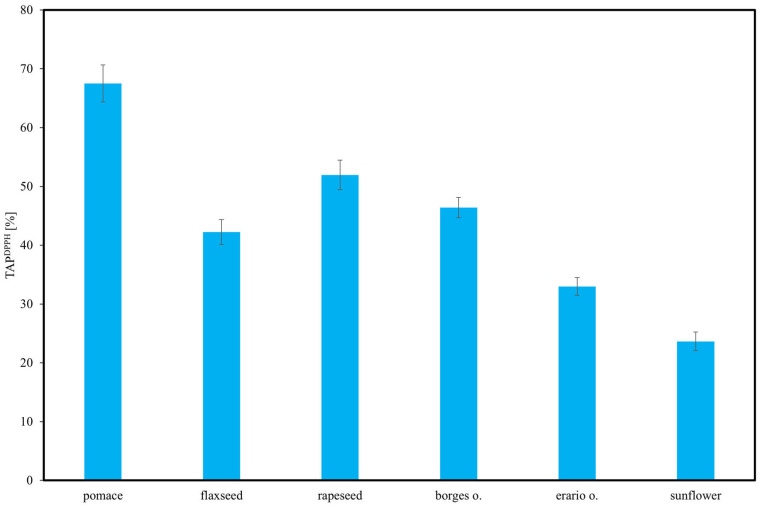
Total antioxidant potential (TAP) of selected oils estimated using 2,2-diphenyl-1-picrylhydrazyl (DPPH) radical scavenging assay. Results are expressed as mean ± SD (*n* = 3).

TAP^DPPH^ of the analyzed oils varied significantly, as illustrated in Figure 7. The highest activity was recorded for rapeseed pomace and rapeseed oil, both exceeding 60%, indicating a high concentration of bioactive compounds with strong antioxidative properties. These results underscore the compositional diversity of edible oils and their varying capacities as sources of natural antioxidants. Our results for the DPPH activity of flaxseed oil (≈45%) are consistent with previous data, confirming its moderate radical scavenging capacity compared to rapeseed or pomace [4]. The application of the DPPH assay ensured consistency with the majority of published reports, while other assays such as FRAP were not considered directly comparable. These findings are consistent with the results obtained from TAP HPLC–ED and other analytical methods. Sunflower oil showed moderate antioxidant activity, lower than rapeseed samples but higher than the remaining oils. Flaxseed oil demonstrated greater TAP^DPPH^ than Borges and Erario olive oils, suggesting a higher content of strong antioxidants. Among all samples, the two olive oils exhibited the lowest antioxidant activity, likely due to their lower levels of tocopherols, polyphenols, or other antioxidative constituents. These results underscore the compositional diversity of edible oils and their varying capacity as sources of natural antioxidants.

### 2.4. TAP^Folin^ Measurements

The Folin–Ciocalteu assay is primarily employed to determine the total polyphenol content in aqueous samples. Due to the strong correlation between polyphenol content and antioxidant capacity, it is also used for estimating TAP, with gallic acid typically serving as the reference standard. However, several limitations must be considered. The assay is highly sensitive to factors such as pH, reaction time, and temperature, which can affect reproducibility [58]. Moreover, it tends to overestimate polyphenol content, as the FC reagent reacts not only with phenolic compounds but also with other reducing agents such as sugars and certain amino acids. Additionally, the method is restricted to aqueous systems, limiting its ability to detect lipophilic phenols unless modified assay versions are employed [59,60].

**Figure 8:**
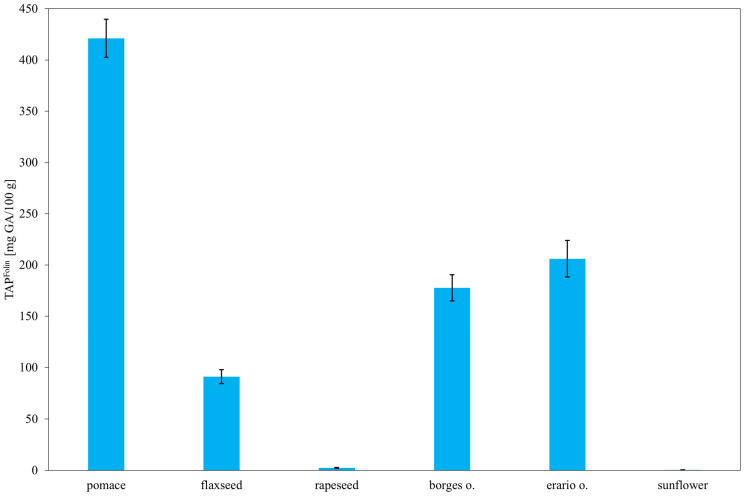
Total antioxidant potential (TAP) of selected oils estimated by the Folin–Ciocalteu assay. Results are expressed as mean ± SD (*n* = 3).

As shown in Figure 8, the highest concentration of polyphenols was observed in rapeseed pomace, exceeding 400 mg gallic acid equivalents (GAE)/100 g. This is consistent with the known composition of rapeseed, which contains significant amounts of phenolic acids such as caffeic, syringic, sinapic, and ferulic acids, along with their derivatives—compounds well-documented for their antioxidant properties. These results suggest that during oil pressing, a substantial portion of phenolic compounds remains in the pomace. In contrast, sunflower oil exhibited the lowest TAP, correlating with Figure 5, where its antioxidant capacity is primarily attributed to tocopherols rather than polyphenols. Rapeseed oil, being a commercially refined product, may have undergone processing steps that remove part of its antioxidant content, which could explain its lower polyphenol concentration compared to pomace. Notably, the two olive oils (Erario and Borges) showed divergent polyphenol contents. Erario exhibited the second-highest GAE value, while Borges had the lowest (excluding results within statistical uncertainty). This discrepancy may be due to differences in geographic origin—Erario is produced in Italy and Borges in Spain—since factors such as soil composition and cultivation practices can significantly affect polyphenol levels. Another likely explanation is the processing method: unrefined, cold-pressed olive oils tend to retain more antioxidants than refined ones. Flaxseed oil, although primarily composed of omega-3 fatty acids, also contains minor amounts of polyphenols, including lignans and phenolic acids [61,62], accounting for its moderate GAE values. These findings are consistent with previous studies [63,64], supporting the conclusion that both composition and processing significantly influence the antioxidant profiles of edible oils.

### 2.5. TAP^ED^ Measurements

**Figure 9:**
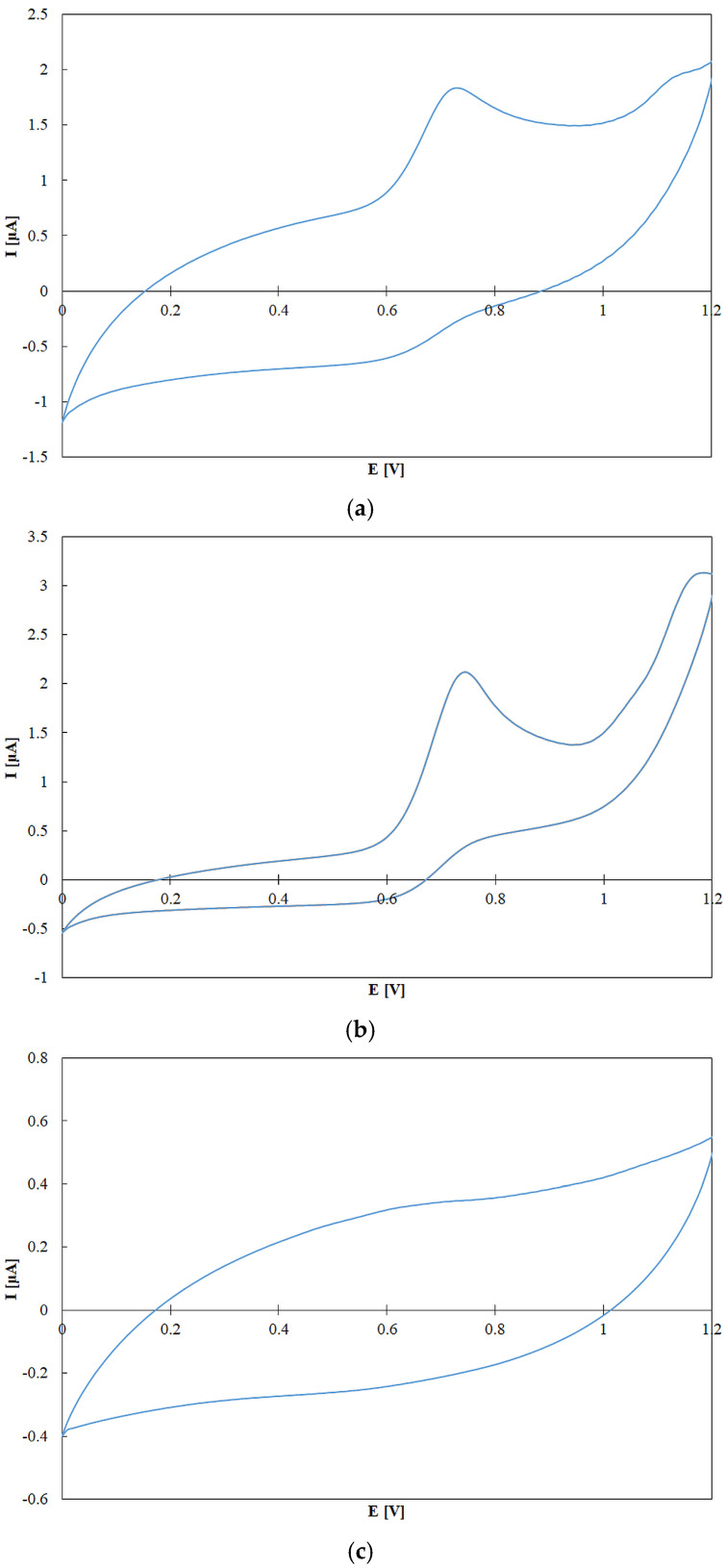
Cyclic voltammograms (CV) of rapeseed pomace (**a**), borges o. (**b**) and sunflower oil (**c**). Experimental conditions: potential range 0–1.2 V; working electrode, glassy carbon (GC); reference electrode, Ag/AgCl; counter electrode, platinum wire; supporting electrode, 0.1 M NaClO_4_ in methanol.

Voltammetry enables direct measurement of TAP in pure compounds, where TAP is assessed based on the redox potential or the position of the voltammetric peak. However, in the case of complex oil samples, which contain various antioxidants in unknown concentrations, these standard parameters cannot be applied in the same manner. In such mixtures, TAP is considered to be directly proportional to the oxidation current (which reflects concentration) and inversely proportional to the applied potential. Figure 9 presents cyclic voltammograms of rapeseed pomace, olive oil, and sunflower oil. A distinct peak at approximately 0.7 V, particularly visible in rapeseed pomace, can be attributed to antioxidant compounds. Although a more detailed comparative analysis would be necessary to precisely identify the individual components responsible for these signals, such identification is not required for the quantitative assessment of TAP. TAP in electrochemical detection (TAP^ED^) is defined as the area under the voltammetric curve [42]. This is calculated by integrating the measured current with respect to the applied potential, factoring in an exponential relationship with the redox potential of the hydroxyl radical. Hydroxyl radical potential was chosen, because it has the highest redox potential in naturally occurring radicals. The TAP^ED^ was calculated using Equation (1).

(1) $$TAP^{ED}=\int_{E_{1}}^{E_{2}}\left[I\left(E\right)-I_{0}\left(E\right)\right]exp^{\left(2.08-E\right)}dE$$

where E—potential, E_1_, E_2_—potential range (0–1.2 V), I—current, I_0_—base current, 2.08—standard reduction potential of the hydroxyl radical vs. Ag/AgCl electrode. 

This approach allows for the estimation of TAP^ED^ by integrating the net oxidation current, weighted by the exponential factor related to antioxidant activity. The choice of 2.08 V as the reference reflects the high oxidative strength of hydroxyl radicals (related to Ag/AgCl electrode), thus enabling meaningful comparisons across antioxidant-rich samples.

**Figure 10:**
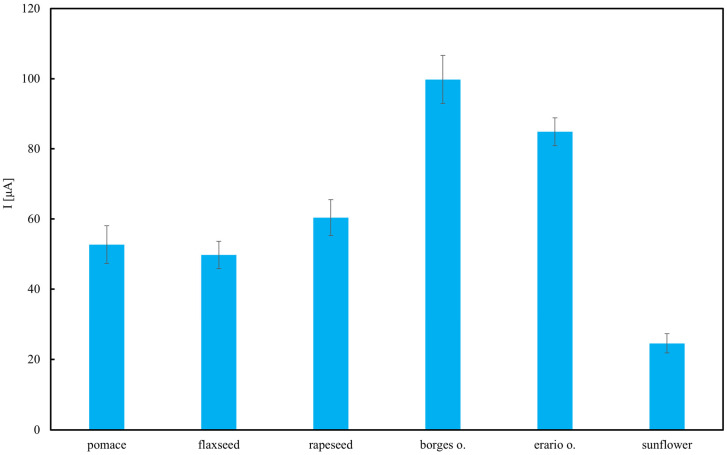
Total antioxidant potential (TAP) of selected oils estimated using cyclic voltammetry (CV). Results are expressed as mean ± SD (*n* = 3).

Figure 10 presents TAP of various edible oils, measured electrochemically and expressed as current (I) in microamperes (µA). The results indicate that the highest antioxidant activity was observed in the olive oils—Borges and Erario—with Borges oil reaching approximately 1.5 µA, followed closely by Erario. This is consistent with literature reports highlighting the high polyphenol content in olive oils, compounds well known for their strong antioxidant properties [65,66]. The slight variation between Borges and Erario may be attributed to differences in cultivar, geographic origin, extraction method, or storage conditions. Rapeseed and flaxseed oils showed moderate TAP values, ranging from 0.7 to 0.85 µA. This suggests that, while they contain antioxidant constituents such as tocopherols and omega-3 fatty acids, their polyphenol levels are lower compared to olive oils. These findings are in agreement with results obtained using the Folin–Ciocalteu assay. Interestingly, rapeseed pomace retains a measurable level of antioxidant activity. This suggests that certain antioxidant compounds, though not necessarily electroactive to the same extent as those in olive oils, persist in the pomace. If only one type of analysis was employed, this result could not be recorded. This is the reason why multiple assays should be employed for reliable antioxidant analysis [67]. The effectiveness of electrochemical methods for detecting antioxidants in complex food matrices has been recently demonstrated, confirming their sensitivity and practical applicability [68]. In contrast, sunflower oil exhibited the lowest antioxidant activity (≈0.3 µA). This may be due to the refining process, which tends to reduce the levels of naturally occurring antioxidants. Although sunflower oil contains vitamin E (primarily α-tocopherol), it lacks significant concentrations of polyphenols, contributing to its lower electrochemical antioxidant capacity.

### 2.6. Statistical Analysis

#### 2.6.1. Two-Way ANOVA (TAP^HPLC^)

For electrochemical detection (HPLC–ED), a two-way ANOVA was performed with *oil type* (6 levels) and *fraction* (3 levels) as factors. Necessary data is presented in Table 1, Table 2, Table 3, Table 4, Table 5 and Table 6. At both applied potentials (0.6 V and 0.8 V), significant main effects of oil and fraction were observed (*p* < 0.001), as well as strong oil × fraction interactions. Post hoc comparisons confirmed that the total fraction was consistently higher than tocopherols or other components, and that pomace again showed the strongest antioxidant response.

Although the Shapiro–Wilk and Levene’s tests (Table 7) indicated partial deviations from normality and homogeneity of variances, it is well established that ANOVA is robust to moderate violations of these assumptions, particularly when group sizes are comparable. Moreover, the large effect sizes and consistent post hoc results support the reliability of the findings; nevertheless, the limitations associated with these assumption breaches should be acknowledged when interpreting the outcomes.

#### 2.6.2. One-Way ANOVA (TAP^DPPH^, TAP^Folin^, TAP^CV^)

The results for one-way ANOVA calculations are presented in Table 8, Table 9, Table 10, Table 11, Table 12 and Table 13. For the DPPH, Folin–Ciocalteu and CV methods, one-way ANOVA revealed highly significant differences among oils (all *p* < 0.001). Tukey’s post hoc tests showed distinct groupings, indicating that rapeseed pomace consistently exhibited the highest antioxidant potential, while sunflower oil was at the lowest end. Intermediate results were observed for flaxseed and rapeseed, whereas olive oils were positioned in the mid-range.

#### 2.6.3. Antioxidant Reactivity Index

**Figure 11:**
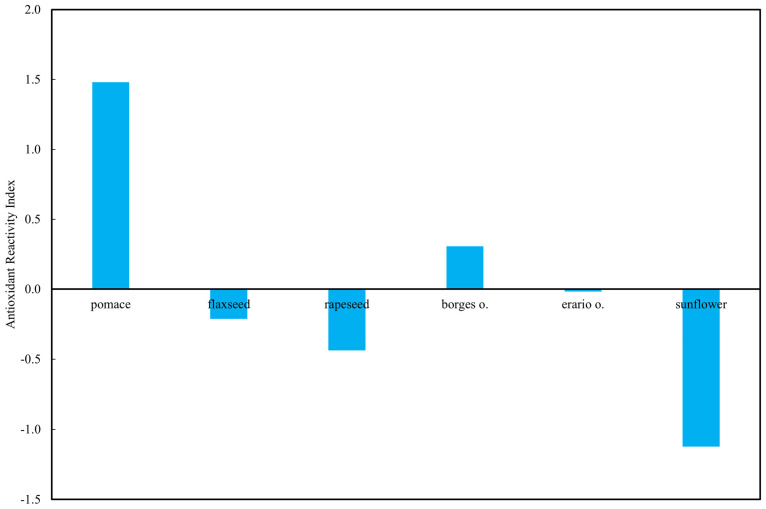
Antioxidant Reactivity Indexes (ARI) of selected oils.

The standardized ARI highlighted distinct differences among the tested oils (Figure 11). Rapeseed pomace exhibited by far the highest ARI, indicating consistently elevated antioxidant activity across all applied methods. In contrast, sunflower oil showed the lowest ARI value, clearly below zero, reflecting uniformly weak responses irrespective of the assay. The two commercial olive oils presented intermediate behavior: Borges olive oil reached a slightly positive ARI, while Erario olive oil remained close to zero, suggesting method-dependent variability. Flaxseed and rapeseed oils also displayed negative ARI values, underscoring their relatively lower antioxidant reactivity compared to pomace.

This pattern emphasizes the strong antioxidant potential retained in rapeseed pomace, a by-product of oil pressing, whereas sunflower oil, despite its common use, appears consistently less effective in terms of radical-scavenging and redox-active components.

#### 2.6.4. Principal Component Analysis (PCA)

PCA reduced the five analytical methods to two principal components, which together explained 92% of the total variance. Dimension 1 (70.3%) captured the common signal shared by HPLC–ED (0.6 V, 0.8 V), DPPH, and Folin–Ciocalteu assays, reflecting overall antioxidant activity as determined by radical-scavenging and redox-sensitive methods. Dimension 2 (21.7%) was dominated by CV, confirming that this electrochemical approach contributes information largely independent from the other assays.

**Figure 12:**
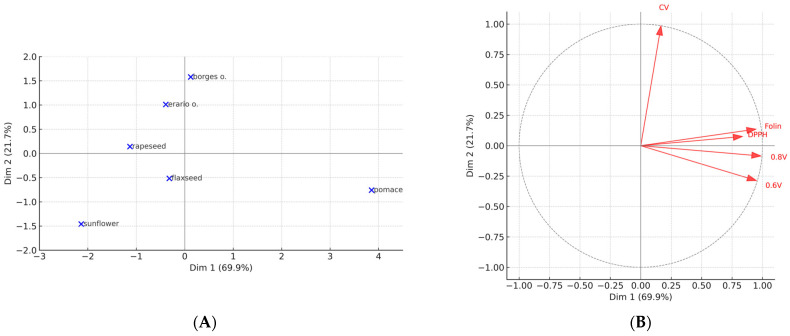
Principal component analysis (PCA) of antioxidant assays in oils. (**A**) Projection of oil samples on the first two principal components. (**B**) Correlation circle showing the contribution of antioxidant assays (HPLC–ED at 0.6 V and 0.8 V, DPPH, Folin–Ciocalteu, and CV) to the principal components.

The biplot (Figure 12A,B) provided clear sample separation. Rapeseed pomace was strongly distinguished along Dimension 1, indicating uniformly high antioxidant reactivity across all methods. Sunflower oil, in contrast, was positioned at the opposite end of Dimension 1, confirming its consistently weak antioxidant responses. The two commercial olive oils (Borges and Erario) clustered together, suggesting a similar antioxidant profile with moderate values across most methods. Seed oils (rapeseed and flaxseed) formed a separate cluster, closer to the origin, reflecting intermediate reactivity.

The variable correlation plot further illustrated the relationships among assays. HPLC–ED (both potentials) and Folin were highly collinear, with DPPH also positioned in their proximity, indicating that these assays probe overlapping aspects of antioxidant potential. By contrast, CV was projected orthogonally to this cluster, confirming its unique analytical contribution and justifying its inclusion as a complementary method.

Together, the PCA results emphasize that rapeseed pomace stands out as a source of redox-active compounds, while sunflower oil consistently groups as the weakest. Moreover, the separation of assay clusters highlights the importance of combining methods with different mechanistic bases in order to obtain a more comprehensive picture of antioxidant capacity.

#### 2.6.5. Correlation and Reliability

**Figure 13:**
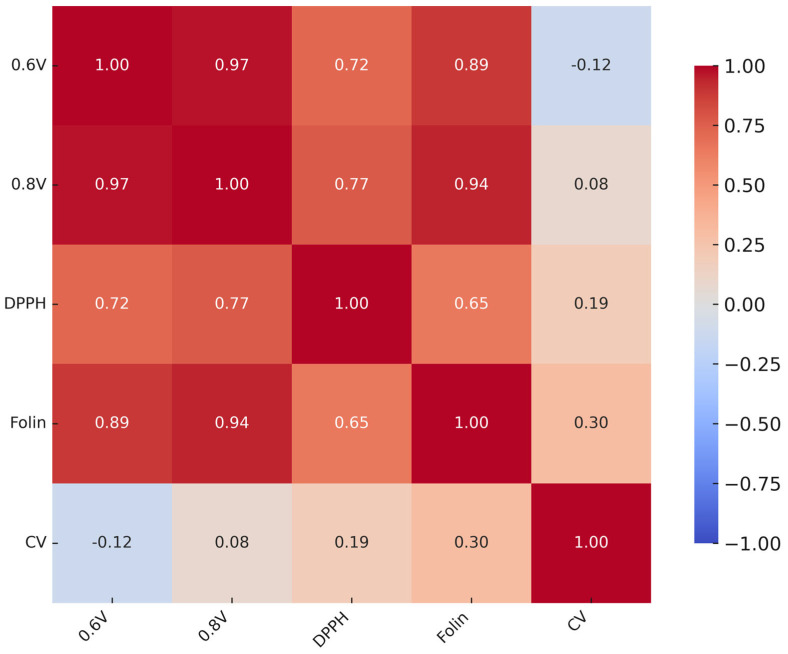
Pearson correlation matrix of antioxidant assays.

Correlation analysis (Figure 13) revealed strong associations between HPLC–ED assays (*r* > 0.95) and moderate-to-strong correlations with the Folin (*r* = 0.83–0.90). In contrast, CV showed weak or negative correlations with the other methods. Cronbach’s *α* (0.854) indicated high overall consistency among assays, whereas the Friedman test confirmed significant differences between methods (*χ*^2^ = 16.1, *df* = 4, *p* = 0.003).

Taken together, these findings suggest that while most methods provide coherent information on antioxidant reactivity, CV measures a distinct aspect of antioxidant behavior.

### 2.7. Limitations

This study is presented as preliminary research, with the main objective of demonstrating feasibility of multi-method antioxidant assessment rather than providing exhaustive quantitative evaluation. The analysis was based on triplicate measurements (*n* = 3) for each determination, which allowed robust statistical evaluation and minimized random variability. Nevertheless, several limitations should be acknowledged. First, the dataset was restricted to oils available on the local Polish market, which reflects real-world consumer conditions but may reduce generalizability. Second, although multiple analytical assays were applied (HPLC–ED, CV, DPPH, FC), all are chemical in vitro methods and cannot fully capture the biological relevance of antioxidant activity. The inclusion of DPPH and Folin–Ciocalteu, despite their well-known limitations and lack of specificity, was intentional: DPPH remains the most widely applied radical scavenging assay, while Folin–Ciocalteu is a routine proxy for total phenolics. Both methods, although imperfect, provide a useful comparative background. Importantly, relying on assays based on the same underlying mechanism can severely limit interpretation, as they often probe distinct facets of antioxidant reactivity [67]. This was most evident in the divergent results obtained for olive oil and rapeseed pomace. Therefore, our study deliberately combined chromatographic, spectrophotometric, and electrochemical approaches to capture complementary information. Finally, while our findings indicate that rapeseed pomace retains substantial antioxidant potential and could be valorized as a functional food, dietary supplement, or feed additive, this hypothesis requires further validation. Specifically, in vitro digestion models and in vivo studies would be necessary to assess the bioavailability, stability, and physiological impact of its antioxidant constituents on humans and animals.

## 3. Materials and Methods

### 3.1. Reagents

HPLC-grade methanol (≥99.9%), camphorsulfonic acid, DPPH, Folin–Ciocalteu reagent were obtained from Sigma-Aldrich (Saint Louis, MO, USA), mixed tocopherol standards (total concentration: 726 mg/mL; α-tocopherol: 143 mg/mL; β-tocopherol: 11 mg/mL; γ-tocopherol: 444 mg/mL; δ-tocopherol: 128 mg/mL) and sodium perchlorate (NaClO_4_) were procured from Sigma-Aldrich (Saint Louis, MO, USA). Sodium carbonate (Na_2_CO_3_) was obtained from Chempur (Piekary Śląskie, Poland). All chemicals were analytical grade and used without further purification.

Water was triple-distilled using a quartz distillation apparatus (Heraeus Quarzglas, Destamat, Germany). Samples for HPLC–ED analysis were filtered through a 0.22 μm membrane filter (Millipore, Bedford, MA, USA) prior to use.

### 3.2. Materials

Commercial edible oils were sourced from supermarkets in Poland, including: Erario (Italy, extra virgin olive oil), Borges (Spain, extra virgin olive oil), Mazowiecki (Poland, rapeseed oil, refined), Niharti (UK, flaxseed oil, refined), and Bartek (Poland, sunflower oil, refined). Rapeseed pomace was obtained from MGW-Krzysztof Kurciński Sp. z o.o. (Bytom, Poland).

### 3.3. Sample Preparation

In this study, we evaluated the feasibility of extracting oils with methanol using an ultrasonic bath. Briefly, 0.2 g of oil was placed in a 10 mL volumetric flask filled with methanol, and the flask was subjected to ultrasonic treatment for approximately 30 min. The resulting solution was filtered through a 0.45 µm nylon membrane filter (Millipore, Bedford, MA, USA) and analyzed by HPLC [69].

Calibration curve for gallic acid was constructed in the range 0.001–0.005 mg/mL, showing excellent linearity (R^2^ = 0.994; y = 0.9941x). Results were expressed as mg GAE/100 g of oil.

### 3.4. Apparatus

HPLC analysis was conducted using a chromatographic system comprising an Interface Box, 4-channel Smartline Manager 5000 with Degasser K-5004, Solvent Organizer K-1500, Dynamic Mixing Chamber, HPLC Pump Smartline 1000, UV/Vis Diode Array Detector Smartline 2600, 20 μL D-14163 injection valve, and Smartline 4000 Column Thermostat; all components were from Knauer GmbH (Knauer, Berlin, Germany). An amperometric detector (Recipe, Berlin, Germany; ClinLab EC3000) equipped with a glassy carbon (GC) working electrode, Ag/AgCl reference electrode, and auxiliary electrode (cell body) was used, alongside an autosampler (Smartline-3900). Separation was performed on a COSMOSIL 5C18-MS-II column (250 × 4.6 mm I.D., 5 µm particle size; Knauer, Berlin, Germany).

pH measurements were carried out using a pH meter OP-208/1 (Radelkis, Budapest, Hungary) equipped with an OSH 10-10 electrode (Metron, Aargau, Switzerland).

Photometric assays (DPPH and FC) were performed using a Helios Epsilon spectrophotometer (Thermo Fisher Scientific, Waltham, MA, USA) and a DU68 spectrophotometer (Beckman, Brea, CA, USA).

Electrochemical measurements were performed with an Autolab PGSTAT20 potentiostat/galvanostat (Eco Chemie, Utrecht, Netherlands) using a three-electrode system. The working electrode was a 2 mm diameter glassy carbon disk, polished prior to each measurement with a 0.05 µm alumina aqueous suspension on a polishing pad, followed by rinsing with water. A platinum wire served as the auxiliary electrode, and a saturated Ag/AgCl electrode (3 mol/L KCl) functioned as the reference electrode. Data acquisition and control were managed via GPES v4.9 software on an IBM PC.

### 3.5. Procedures

#### 3.5.1. HPLC Measurements

Chromatographic analyses were performed at a flow rate of 1.0 mL/min. The column was equilibrated at 20 °C by passing the mobile phase through it for 1 h prior to sample injection. The mobile phase consisted of 0.1 M NaClO_4_ in methanol/water (99:1, *v*/*v*). Samples (20 µL) were injected using an autosampler. All measurements were conducted at 20 °C. Electrochemical detector signals were continuously monitored and recorded by the computer. The working electrode potential was set at 0.6 V and 0.8 V to differentiate between strong and weak antioxidants. Each analysis lasted 20 min. TAP was assessed by calculating the total peak area on the chromatogram corresponding to retention times (tR) of up to 20 min at the specified electrode potentials.

Calibration curves for α-, δ-, and β+γ-tocopherols (the latter quantified jointly due to co-elution) were established in the range 0.2–100 µg/mL. Linear regression demonstrated good correlation, with *R*^2^ = 0.9396 for δ-tocopherol (y = 71.25x + 83.755), *R*^2^ = 0.9724 for β+γ-tocopherols (y = 281.39x + 225.89), and *R*^2^ = 0.9826 for α-tocopherol (y = 90.481x + 59.462). Slight deviations from linearity at higher concentrations (>60 µg/mL) were attributed to detector saturation. LOD and LOQ, calculated as 3.3σ/s and 10σ/s, respectively, were in the range of 2–4 µg/mL (LOD) and 6–12 µg/mL (LOQ) depending on the homolog. Precision, assessed by replicate injections at 10 µg/mL, did not exceed 3% RSD for peak areas and 0.5% for retention times. These results confirm the suitability of the HPLC–ED method for quantitative tocopherol determination in oils and rapeseed pomace.

#### 3.5.2. DPPH Assay

The measurement of TAP related to the DPPH radical (TAP^DPPH^) involved mixing the sample solution and methanol to a total volume of 1 mL with 1 mL of 1 mM DPPH solution in methanol. The mixture was vigorously shaken and incubated in the dark at room temperature for 30 min. Subsequently, the absorbance was measured at 517 nm. 

The DPPH assay was selected because it is one of the most widely applied and standardized methods for evaluating antioxidant activity in edible oils, ensuring reliable comparison with published data. Alternative assays such as FRAP or superoxide radical scavenging were not included, as they require different experimental conditions and reagents, which would limit direct comparability.

#### 3.5.3. Total Polyphenol Content

Polyphenols content was examined using a modified Folin–Ciocalteu method [70]. 50 µL of the sample (initial concentration 0.02 g/mL) was added to a 10 mL volumetric flask, followed by 1 mL of Folin–Ciocalteu reagent. After 3 min, 4 mL of 20% Na_2_CO_3_ solution was added, and the volume was adjusted to the mark with distilled water. The mixture was incubated in the dark for 30 min to allow the reaction to stabilize. Absorbance was then measured at 765 nm against a blank containing all reagents except the sample. Results were expressed as gallic acid equivalents (GAE, mg GAE/100 g of oil).

#### 3.5.4. Cyclic Voltammetry

Electrochemical measurements by cyclic voltammetry were conducted in a 10 mL electrochemical cell using 0.1 M NaClO_4_ in methanol as the supporting electrolyte. To prevent irreversible adsorption of sample components on the working electrode, 1 mM camphorsulfonic acid was added. Measurements were performed at a scan rate of 200 mV/s over a potential range of 0 to 1.2 V (positive direction). Prior to each measurement, the working electrode was mechanically cleaned using diamond paste followed by polishing with a slurry of water and 1 µm alumina particles. All solutions were degassed by purging with argon for 5 min and allowed to equilibrate for 10 min before measurements.

### 3.6. Statistical Analysis

All determinations of TAP were performed in triplicate (*n* = 3). Results are presented as mean values ± standard deviation (SD).

For the HPLC–ED method, two-way ANOVA was applied with oil type (six levels) and fraction (total, tocopherols, others; three levels) as fixed factors, separately for 0.6 V and 0.8 V potentials. For spectrophotometric (DPPH, Folin–Ciocalteu) and electrochemical (CV) assays, one-way ANOVA was used to compare antioxidant activity across oil types. Significant effects were further explored using Tukey’s post hoc tests.

Assumptions of ANOVA were verified. Homogeneity of variance was assessed with Levene’s and Shapiro–Wilk tests; although deviations were detected in some cases, ANOVA was retained as the primary model due to its robustness to moderate violations in balanced designs.

To enable cross-method comparison, raw TAP values obtained from all assays were standardized (*z*-scores), and an Antioxidant Reactivity Index (ARI) was calculated as the mean of standardized values for each oil. This integrated index reflects the relative antioxidant capacity independently of measurement scale.

Relationships among methods were further examined using Pearson correlation coefficients. Principal component analysis (PCA) with varimax rotation was performed on standardized values to visualize similarities between assays and oil samples. The number of components was determined using eigenvalues > 1.

All analyses were carried out in Jamovi 2.6.26, with statistical significance set at *p* < 0.05, along with statistic-related graphic (biplot) generation.

## 4. Conclusions

This study demonstrates that combining chromatographic, electrochemical, and spectrophotometric methods can provide complementary insights into the antioxidant composition of edible oils and rapeseed pomace. Triplicate measurements and cross-validation across independent assays improved the reliability of the findings. Rapeseed pomace exhibited high tocopherol content and strong antioxidant potential, supporting its valorization as a functional byproduct for use in foods, nutraceuticals, and animal feed. The differences observed between assays (HPLC–ED, CV, DPPH, FC) reflect their distinct detection principles and underline the importance of multi-method strategies for antioxidant evaluation. In addition, the study demonstrates the feasibility of methanol extraction as a practical alternative to classical saponification for tocopherol determination. Overall, these findings highlight rapeseed pomace as a promising functional ingredient, while providing a methodological framework for antioxidant assessment. Future research should include a wider range of oils, more commercial brands, and in vitro/in vivo studies to confirm the bioavailability and health benefits of rapeseed pomace antioxidants.

## Figures and Tables

**Table 1:** Two-way ANOVA results for Total Antioxidant Capacity (TAP) values determined by HPLC at 0.6 V.

Source	*SS*	*df*	*MS*	*F*	*p*
Oil	408.7	5	81.73	222.4	<0.001
Fraction	95.9	2	47.97	130.5	<0.001
Oil × Fraction	106.1	10	10.61	28.9	<0.001
Residuals	13.2	36	0.37	–	–

*SS*—sum of squares; *df*—degrees of freedom; *MS*—mean square; *F*—Fisher’s test statistic; *p*—significance level.

**Table 2:** Tukey’s post hoc comparisons of oils for TAP values determined by HPLC at 0.6 V.

Comparison	Mean Difference	*p*
Pomace vs. Flaxseed	+6.37	<0.001
Pomace vs. Rapeseed	+8.37	<0.001
Pomace vs. Borges o.	+6.73	<0.001
Pomace vs. Erario o.	+7.27	<0.001
Pomace vs. Sunflower	+7.24	<0.001
Flaxseed vs. Rapeseed	+1.99	<0.001
Flaxseed vs. Erario o.	+0.90	0.036
Flaxseed vs. Sunflower	+0.87	0.047
Rapeseed vs. Borges o.	−1.64	<0.001
Rapeseed vs. Erario o.	−1.10	0.006
Rapeseed vs. Sunflower	−1.13	0.004

*p*—significance level.

**Table 3:** Tukey’s post hoc comparisons of fractions for TAP values determined by HPLC at 0.6 V.

Comparison	Mean Difference	*p*
Total vs. Tocopherols	+2.78	<0.001
Total vs. Others	+2.88	<0.001
Tocopherols vs. Others	+0.10	0.878

*p*—significance level.

**Table 4:** Two-way ANOVA results for the effect of oil type and fraction on TAP values determined by HPLC at 0.8 V.

Source	*SS*	*df*	*MS*	*F*	*p*
Oil	514.4	5	102.888	115.8	<0.001
Fraction	234.2	2	117.111	131.9	<0.001
Oil × Fraction	170.2	10	17.017	19.2	<0.001
Residuals	32.0	36	0.888	–	–

*SS*—sum of squares; *df*—degrees of freedom; *MS*—mean square; *F*—Fisher’s test statistic; *p*—significance level.

**Table 5:** Tukey’s post hoc comparisons of oils for TAP values determined by HPLC at 0.8 V.

Comparison	Mean Difference	*p*
Pomace vs. Flaxseed	6.20	<0.001
Pomace vs. Rapeseed	8.87	<0.001
Pomace vs. Borges o.	6.65	<0.001
Pomace vs. Erario o.	6.89	<0.001
Pomace vs. Sunflower	9.53	<0.001
Flaxseed vs. Rapeseed	2.67	<0.001
Flaxseed vs. Erario o.	3.33	<0.001
Flaxseed vs. Sunflower	−2.22	<0.001
Rapeseed vs. Borges o.	−1.98	0.001
Rapeseed vs. Erario o.	2.88	<0.001
Rapeseed vs. Sunflower	2.63	<0.001

*p*—significance level.

**Table 6:** Tukey’s post hoc comparisons of fractions for TAP values determined by HPLC at 0.8 V.

Comparison	Mean Difference	*p*
Total—Tocopherols	4.89	<0.001
Total—Others	3.70	<0.001
Tocopherols—Others	−1.20	0.001

*p*—significance level.

**Table 7:** Results of Levene and the Shapiro–Wilk tests.

Potential	Levene Variances Test	Shapiro–Wilk Normality Test
0.6 V	*p* = 0.007	*p* = 0.035
0.8 V	*p* = 0.015	*p* = 0.269

**Table 8:** One-way ANOVA results for the DPPH assay.

ANOVA	*SS*	*df*	*MS*	*F*	*p*
Between oils	3479.8	5	695.96	97.3	<0.001
Residuals	85.8	12	7.15		
Total	3565.6	17			

*SS*—sum of squares; *df*—degrees of freedom; *MS*—mean square; *F*—Fisher’s test statistic; *p*—significance level.

**Table 9:** Tukey’s HSD post hoc comparisons for the DPPH assay.

Comparisons	Mean Difference	*p*
Pomace—Flaxseed	25.27	<0.001
Pomace—Rapeseed	15.56	<0.001
Pomace—Borges o.	21.11	<0.001
Pomace—Erario o.	34.52	<0.001
Pomace—Sunflower	43.86	<0.001
Flaxseed—Rapeseed	−9.70	0.008
Flaxseed—Erario o.	9.25	0.011
Flaxseed—Sunflower	18.59	<0.001
Rapeseed—Erario o.	18.95	<0.001
Rapeseed—Sunflower	28.30	<0.001
Borges o.—Erario o.	13.41	<0.001
Borges o.—Sunflower	22.75	<0.001
Erario o.—Sunflower	9.35	0.011

*p*—significance level.

**Table 10:** One-way ANOVA results for the Folin–Ciocalteu assay.

ANOVA	*SS*	*df*	*MS*	*F*	*p*
Between oils	375,219	5	75,044	343	<0.001
Residuals	2628	12			
Total	377,847	17			

*SS*—sum of squares; *df*—degrees of freedom; *MS*—mean square; *F*—Fisher’s test statistic; *p*—significance level.

**Table 11:** Tukey’s HSD post hoc comparisons for the Folin–Ciocalteu assay.

Comparison	Mean Difference	*p*
Pomace—Flaxseed	+329.9	<0.001
Pomace—Rapeseed	+418.8	<0.001
Pomace—Borges o.	+243.5	<0.001
Pomace—Erario o.	+215.1	<0.001
Pomace—Sunflower	+420.7	<0.001
Flaxseed—Rapeseed	+88.9	<0.001
Flaxseed—Borges o.	−86.5	<0.001
Flaxseed—Erario o.	−114.9	<0.001
Flaxseed—Sunflower	+90.8	<0.001
Rapeseed—Borges o.	−175.4	<0.001
Rapeseed—Erario o.	−203.8	<0.001
Rapeseed—Sunflower	+1.9	1.000
Borges o.—Erario o.	−28.4	0.247
Borges o.—Sunflower	+177.3	<0.001
Erario o.—Sunflower	+205.7	<0.001

*p*—significance level.

**Table 12:** One-way ANOVA results for the CV assay.

ANOVA	*SS*	*df*	*MS*	*F*	*p*
Between oils	10,777	5	2155.5	61.4	<0.001
Residuals	421	12	35.1		
Total	10,818	17			

*SS*—sum of squares; *df*—degrees of freedom; *MS*—mean square; *F*—Fisher’s test statistic; *p*—significance level.

**Table 13:** Tukey’s HSD post hoc comparisons for the CV assay.

Comparison	Mean Difference	*p*
Pomace—Flaxseed	+2.96	0.988
Pomace—Rapeseed	−7.68	0.621
Pomace—Borges o.	−47.08	<0.001
Pomace—Erario o.	−32.19	<0.001
Pomace—Sunflower	+28.14	<0.001
Flaxseed—Rapeseed	−10.64	0.305
Flaxseed—Borges o.	−50.04	<0.001
Flaxseed—Erario o.	−35.15	<0.001
Flaxseed—Sunflower	+25.18	0.002
Rapeseed—Borges o.	−39.40	<0.001
Rapeseed—Erario o.	−24.51	0.003
Rapeseed—Sunflower	+35.82	<0.001
Borges o.—Erario o.	+14.89	0.080
Borges o.—Sunflower	+75.22	<0.001
Erario o.—Sunflower	+60.33	<0.001

*p*—significance level.

## Data Availability

A preprint version of this manuscript has previously been published on SSRN (DOI: http://dx.doi.org/10.2139/ssrn.5369111) The present version includes full statistical analyses, ex-tended discussion, and revised conclusions. Dataset available on request from the authors.

## Abbreviations

The following abbreviations are used in this manuscript:

AAPH	2,2′-Azobis(2-methylpropionamidine) dihydrochloride
ABAP	2,2′-Azobis(2-amidinopropane) dihydrochloride
ABTS	2,2′-Azino-bis(3-ethylbenzothiazoline-6-sulfonic acid
AIBN	Azobisisobutyronitrile
AMVN	2,2′-Azobis(2,4-dimethylvaleronitrile)
ARI	Antioxidant Reactivity Index
CUPRAC	Cupric Ion Reducing Antioxidant Capacity
CV	cyclic voltammetry
DMPD	N,N-dimethyl-p-phenylenediamine
DPPH	2,2-Diphenyl-1-picrylhydrazyl
DPV	Differential Pulse Voltammetry
FC	Folin–Ciocalteu
FRAP	Ferric Reducing Antioxidant Power
GAE	Gallic Acid Equivalent
GC	glassy carbon
HAT	Hydrogen Atom Transfer
HPLC-ED	High-Performance Liquid Chromatography with Electrochemical Detection
LOD	Limit of Detection
LOQ	Limit of Quantification
ORAC	Oxygen Radical Absorbance Capacity
PCA	Principal Component Analysis
RSD	Relative Standard Deviation
SD	Standard Deviation
SET	Single Electron Transfer
TAP	Total Antioxidant Potential
TEAC	Trolox Equivalent Antioxidant Capacity
